# The PDGFRβ/ERK1/2 pathway regulates CDCP1 expression in triple-negative breast cancer

**DOI:** 10.1186/s12885-018-4500-9

**Published:** 2018-05-23

**Authors:** Luca Forte, Federica Turdo, Cristina Ghirelli, Piera Aiello, Patrizia Casalini, Marilena Valeria Iorio, Elvira D’Ippolito, Patrizia Gasparini, Roberto Agresti, Beatrice Belmonte, Gabriella Sozzi, Lucia Sfondrini, Elda Tagliabue, Manuela Campiglio, Francesca Bianchi

**Affiliations:** 1 Molecular Targeting Unit, Fondazione IRCCS Istituto Nazionale dei Tumori, 20133 Milan, Italy; 2 Tumor Genomics Unit, Fondazione IRCCS Istituto Nazionale dei Tumori, 20133 Milan, Italy; 3 Start Up Unit, Fondazione IRCCS Istituto Nazionale dei Tumori, 20133 Milan, Italy; 4 Division of Surgical Oncology, Breast Unit, Fondazione IRCCS Istituto Nazionale dei Tumori, 20133 Milan, Italy; 5 Tumor Immunology Unit, Department of Health, Human Pathology Section, University of Palermo, Palermo, Italy; 6 Dipartimento di Scienze Biomediche per la Salute, Università degli Studi di Milano, via Mangiagalli 31, 20133 Milan, Italy

**Keywords:** TNBC, CDCP1, PDGFRβ, FISH, ERK1/2, PDGF-BB, IHC

## Abstract

**Background:**

CDCP1, a transmembrane protein with tumor pro-metastatic activity, was recently identified as a prognostic marker in TNBC, the most aggressive breast cancer subtype still lacking an effective molecular targeted therapy. The mechanisms driving CDCP1 over-expression are not fully understood, although several stimuli derived from tumor microenvironment, such as factors present in Wound Healing Fluids (WHFs), reportedly increase CDCP1 levels.

**Methods:**

The expression of CDCP1, PDGFRβ and ERK1/2cell was tested by Western blot after stimulation of MDA-MB-231 cells with PDGF-BB and, similarly, in presence or not of ERK1/2 inhibitor in a panel of TNBC cell lines. Knock-down of PDGFRβ was established in MDA-MB-231 cells to detect CDCP1 upon WHF treatment. Immunohistochemical staining was used to detect the expression of CDCP1 and PDGFRβ in TNBC clinical samples.

**Results:**

We discovered that PDGF-BB-mediated activation of PDGFRβ increases CDCP1 protein expression through the downstream activation of ERK1/2. Inhibition of ERK1/2 activity reduced per se CDCP1 expression, evidence strengthening its role in CDCP1 expression regulation. Knock-down of PDGFRβ in TNBC cells impaired CDCP1 increase induced by WHF treatment, highlighting the role if this receptor as a central player of the WHF-mediated CDCP1 induction. A significant association between CDCP1 and PDGFRβ immunohistochemical staining was observed in TNBC specimens, independently of CDCP1 gene gain, thus corroborating the relevance of the PDGF-BB/PDGFRβ axis in the modulation of CDCP1 expression.

**Conclusion:**

We have identified PDGF-BB/PDGFRβ–mediated pathway as a novel player in the regulation of CDCP1 in TNCBs through ERK1/2 activation. Our results provide the basis for the potential use of PDGFRβ and ERK1/2 inhibitors in targeting the aggressive features of CDCP1-positive TNBCs.

**Electronic supplementary material:**

The online version of this article (10.1186/s12885-018-4500-9) contains supplementary material, which is available to authorized users.

## Background

Triple-negative breast cancers (TNBCs) comprise mammary carcinomas that do not express estrogen receptors (ERs), progesterone receptors (PRs), and human epidermal growth factor receptor-2 (HER-2). TNBCs are an aggressive tumor subtype, characterized by a high risk of recurrence within 5 years after diagnosis and a high mortality rate [1, 2]. Due to the lack of specific molecular targets, chemotherapy remains the standard systemic therapy for TNBCs, but it has dissatisfactory long-term results. Recently, we proposed the transmembrane protein CUB domain-containing protein-1 (CDCP1), which is overexpressed in TNBCs and involved in tumor progression, as a new therapeutic target for TNBCs [3]. CDCP1 is a cleavable transmembrane protein that is overexpressed in several types of cancer cells [4–10]. *CDCP1* encodes a 135-kDa protein that is proteolyzed into a cleaved 70-kDa form [11–13], which can homodimerize and initiate prometastatic activity [11, 14].

CDCP1 increases the migration and invasiveness of cancer cells and anchorage-independent cell survival [15, 16] through its interaction with important signalling pathways in tumor aggressiveness, such as Akt [11], PKCδ [17], Src [12, 14, 16, 18–20], and Extracellular signal-regulated kinases 1–2 (ERK1/2) [21]. Accordingly, several studies have suggested that the overexpression of this protein in tumors is related to worse outcomes in lung cancer [4], pancreatic cancer [5], renal cell carcinoma [7], ovarian cancer [8], and hepatocellular carcinoma [9]. The mechanisms by which CDCP1 expression is regulated in TNBCs are unknown.

The correlation that we observed between a gain in *CDCP1* copy number and the number of cells that express CDCP1 in TNBCs supports that *CDCP1* polysomy is involved in CDCP1 overexpression in this breast cancer subtype. However, because approximately 50% of TNBC tumors overexpressing CDCP1 lack polysomy, the CDCP1 expression might be regulated by transcriptional and post-translational mechanisms, regardless of a genetic gain (e.g., by influencing the half-life of CDCP1 through EGFR-mediated inhibition of palmitoylation-dependent degradation of CDCP1 [22]). We demonstrate that activation of platelet-derived growth factor receptors beta (PDGFRβ) by PDGF-BB upregulates CDCP1 expression and that ERK1/2 activation is crucial for this upmodulation. Consistently, a significant association between CDCP1 and PDGFRβ expression was observed in TNBC specimens, independent of gains in *CDCP1*, confirming the link between these two molecules.

## Methods

### Cell lines, cultures, and treatments

The human breast cancer cell lines MDA-MB-231 (ATCC® HTB-26™), BT549 (ATCC® HTB122™), HCC1937 (ATCC® CRL 2336™), MDA-MB-468 (ATCC ® HTB-132™), (American Type Culture Collection, Manassas, VA), SUM149, and SUM159 (Asterand Bioscience, Detroit, MI now acquired by BioreclamationIVT, Westbury, NY) were authenticated using a panel of microsatellite markers. Cell lines were maintained at 37 °C in a humidified atmosphere of 5% CO_2_ as previously described in Turdo et al. [3]. For stimulation experiments, MDA-MB-231 cells were starved in serum-free medium for 24 h and then treated for 48 h with a pool of 5 WHFs at a final concentration of 5% as described [23] or with PDGF-BB, Mib1b, MCP1, IP10, Il1ra, Il1b, G-CSF, Il8, Il6, EGF, FGF, Heregulin, PDGF-AA, PDGF-AB (PeproTech, Rocky Hill, NJ) at 50 ng/mL. Cells were treated in indicated experiments with c*ycloheximide* (1 μM) or UO126 (2 μM), both of which were dissolved in DMSO (maximum concentration 0.1%) (Sigma-Aldrich).

### Antibodies

FACS analysis was performed with Alexa Fluor® 647 anti-human CD318 (CDCP1) (BioLegend, San Diego, CA). Biochemical analyses were performed using rabbit polyclonal antibodies against CDCP1, phospho-CDCP1 (Tyr734), p44/42 MAPK (ERK1/2), phospho-p44/42 MAPK (ERK1/2) (Thr202/Tyr204) (Cell Signaling, Danvers, MA), and PDGFRβ (Santa Cruz Biotechnology, Dallas, TX) or mouse monoclonal anti-phospho-PDGFRβ (Tyr751) (clone 88H8) (Cell Signaling); polyclonal anti-rabbit or -mouse IgG (GE Healthcare, Chicago, IL) was the secondary antibody. Actin was revealed by probing with peroxidase-linked mouse monoclonal anti-actin (Sigma-Aldrich).

### Western blot

To prepare crude cell lysates, cells were processed as described [24]. Protein concentrations were determined by Coomassie Plus protein assay (Thermo Fisher Scientific, Waltham, MA). The samples were separated on NuPage SDS-Bis-Tris gels (Thermo Fisher Scientific) and transferred to PVDF membranes (Merck Millipore, Billerica, MA). Signals were detected using ECL reagent (GE Healthcare). Protein expression was normalized to that of actin, and densitometry was performed in Quantity One 4.6.6 (Bio-Rad, Hercules, CA).

### Cytofluorimetric analysis

CDCP1 protein was detected by FACScan analysis by staining cells with PE anti-human CD318 (CDCP1) Antibody (BioLegends). Cells not stained with antibody were used as controls. The gates were set based on light scatter properties after debris and doublet exclusion; a representative gating strategy is shown in Additional file 1: Figure S1. Samples were analyzed using a FACSCalibur flow cytometer (BD Bioscineces) and FlowJo software (TreeStar).

### Knockdown of PDGFRβ by siRNA transfection

To knock down PDGFRβ, cells were transfected with 100 nM of specific silencer siRNA (ID s10242) or a N.1 negative control siRNA (Thermo Fisher Scientific) using RNAiMAX (Life Technologies), harvested at 48 h post-transfection, and examined for protein expression by western blot.

### Patients

Samples from 65 TNBC patients diagnosed between August 2002 and February 2007 were collected in our institute (Fondazione IRCCS Istituto Nazionale dei Tumori) [3, 25].

### Immunohistochemistry

Expression of CDCP1 and PDGFRβ was analyzed by IHC in consecutive 2-μm formalin-fixed, paraffin-embedded (FFPE) tumor sections, using rabbit polyclonal anti-CDCP1 (1:50) (PA5–17245, Thermo Fisher Scientific) and rabbit anti-human PDGFRβ (1:200) (Y92, Abcam), respectively. Antigen retrieval was performed by heating the sections for 5 min at 96 °C in 10 mM citrate buffer, pH 6.0. Staining was visualized using streptavidin-biotin-peroxidase (Dako, Agilent Technology, Santa Clara, CA) and 3,3′-diaminobenzidine (DAB; brown signal) (Dako), and the sections were counterstained with hematoxylin. Images were acquired by ECLIPSE TE2000-S inverted microscope (Nikon Instruments, Melville, NY) at 20X and 40X magnification. The reactivity of anti-CDCP1 and anti-PDGFRβ was considered to be positive per Turdo 2016 and D’Ippolito 2016 [3, 25]. Specifically, based on the intensity of PDGFRβ staining in neoplastic cells, we assigned tumors a score of 0 (absence of signal) or 1 (weak to strong cytoplasmic signal and membrane signal). Reactivity of polyclonal anti-CDCP1 was defined as positive when ≥10% of tumor cells showed membrane staining.

### Fluorescence in situ hybridization (FISH)

All FISH analyses were performed in FFPE tissues in areas that were selected by the pathologist as being CDCP1-positive by IHC or, for IHC-negative cases, representative of the tumor. Tumors were classified as positive or negative per Turdo et al. [3].

### Statistical analysis

Relationships between categorical variables were analyzed by Fisher’s exact test. Differences were considered to be significant at *p* ≤ 0.05. All analyses were performed using SAS 9.4 (SAS Institute Inc.).

## Results

### PDGFRβ regulates CDCP1 expression in TNBC cells

To examine the molecules that regulate CDCP1 expression in TNBCs, the TNBC cell line MDA-MB-231 was stimulated for 48 h with various ligands, including growth factors, cytokines, and chemokines in wound healing fluids (WHFs) [26], that we found upregulate CDCP1 robustly [3]. This panel of molecules was chosen from small molecules that are involved in breast cancer progression. Plasma membrane CDCP1 levels were determined by cytofluorimetry, and the percentage of increase in expression following stimulation with each molecule was calculated respect to the maximum increase that was induced by WHF, used as a positive control.

**Fig. 1:**
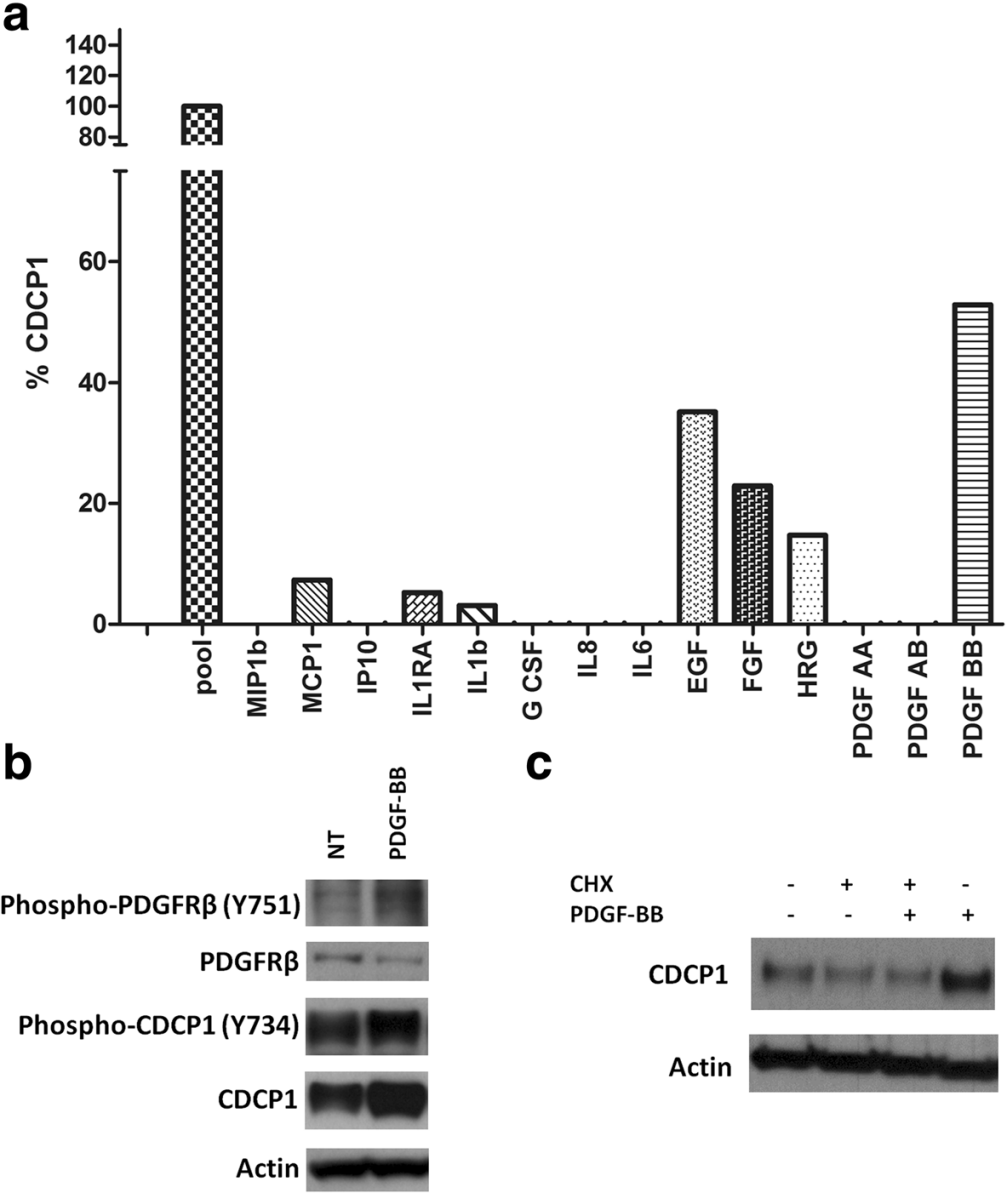
PDGF-BB stimulation upregulates CDCP1 in TNBC cells. **a** CDCP1 in MDA-MB-231 cells treated with various growth factors, cytokines, and chemokines for 48 h was analyzed by FACS and reported as percentage upregulation with respect to the maximum increase induced by WHFs (100%). Representative experiment. **b** WB analysis of CDCP1, phospho-CDCP1 (Y734), PDGFRβ, and phospho-PDGFRβ (Y751) in MDA-MB-231 cells treated with or without PDGF-BB 20 ng/ml for 48 h. The fold-change increase in phospho-CDCP1 and CDCP1, calculated by densitometry, was 1.6 and 1.9, respectively. **c** WB analysis of CDCP1 in MDA-MB-231 cells treated with or without PDGF-BB 20 ng/ml and/or cycloheximide (1 μM) for 24 h. Monoclonal anti-actin was used as the total protein loading control

Regarding growth factors, PDGF-BB was among the strongest inducers of CDCP1 in MDA-MB-231 cells (Fig. 1a). CDCP1 upregulation was also affected by EGF, FGF-basic, and HRG. Of the cytokines and chemokines, except for slight upregulation by MCP1, IL1RA, and IL-1b, none increased CDCP1 levels, suggesting that the regulation of CDCP1 in TNBC cells depends primarily on growth factors. Thus, we focused on the PDGF-BB/PDGFRβ pathway.

By western blot, we confirmed the robust upmodulation of CDCP1 after PDGF-BB stimulation in MDA-MB-231 cells (Fig. 1b). PDGF-BB-induced signalling was initiated by activation of its cognate receptor, PDGFRβ, which results still phosphorylated after 48 h of treatment with PDGF-BB. Consistent with its stimulation, the total level of PDGFRβ decreased compared with unstimulated cells, presumably due to its postactivation protein degradation [27]. Similarly, CDCP1 phosphorylation (Y734) rose after PDGF-BB treatment.

To confirm that the upregulation of CDCP1 protein on PDGF-BB stimulation was attributed to greater CDCP1 translation, MDA-MB-231 cells were stimulated with or without the protein neosynthesis inhibitor *cycloheximide* (CHX). By western blot, no PDGF-BB-induced CDCP1 upmodulation occurred in the presence of CHX, indicating that during PDGF-BB stimulation, the increase in CDCP1 protein is due to protein neosynthesis (Fig. 1c).

**Fig. 2:**
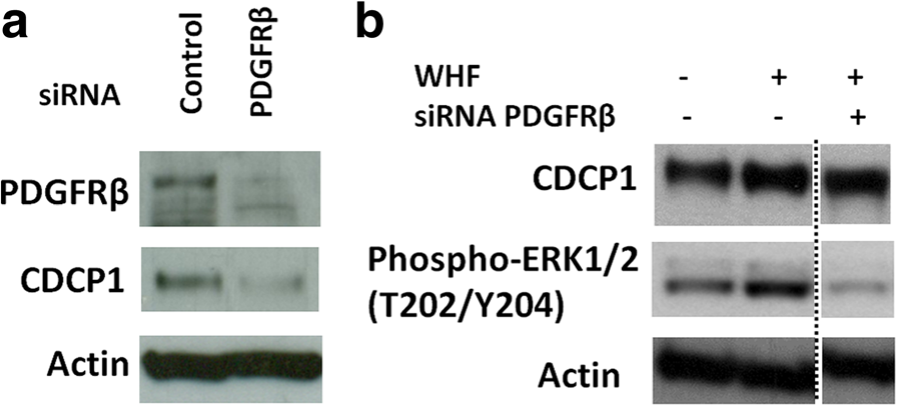
PDGFRβ regulates CDCP1 expression in TNBC cells. **a** WB analysis of PDGFRβ and CDCP1 in MDA-MB-231 cells transfected with 100 nM PDGFRβ siRNA or the appropriate negative control. Cells were harvested at 48 h post-transfection. **b** WB analysis of CDCP1 and phosphoERK1/2 (T202/Y204) in MDA-MB-231 cells transfected with 100 nM PDGFRβ siRNA or the appropriate negative control and with or without 5% WHF in culture medium for 24 h. Dottes lines demarcate juxtaposed images originating from separate lines of the same western blot. The fold-change increase in CDCP1, calculated by densitometry, was 2.3 and 1.6, respectively. Monoclonal anti-actin was used as the total protein loading control

To verify the function of PDGFRβ in the regulation of CDCP1 expression, PDGFRβ was transiently knocked down in MDA-MB-231 cells for 24 h (Fig. 2a). By western blot, CDCP1 declined in PDGFRβ siRNA-treated versus scramble siRNA-treated cells.

To determine whether PDGF-BB participates in the increase in CDCP1 expression after TNBC cell stimulation with WHFs, PDGFRβ was transiently knocked down in MDA-MB-231 cells, which were then stimulated with WHFs for 24 h, 24 h after siRNA transfection (Fig. 2b). In knockdown cells, the increase of CDCP1 was partially impaired on WHF treatment.

PDGFRβ activation triggers several transduction signals, such as the Ras-ERK pathway. ERK1/2 governs the upregulation in CDCP1 following stimulation with growth factors. Notably, ERK was less active in cells in which PDGFRβ was knocked down (Fig. 2b).

### PDGF-BB-induced CDCP1 upregulation depends on the PDGFRβ/ERK axis

**Fig. 3:**
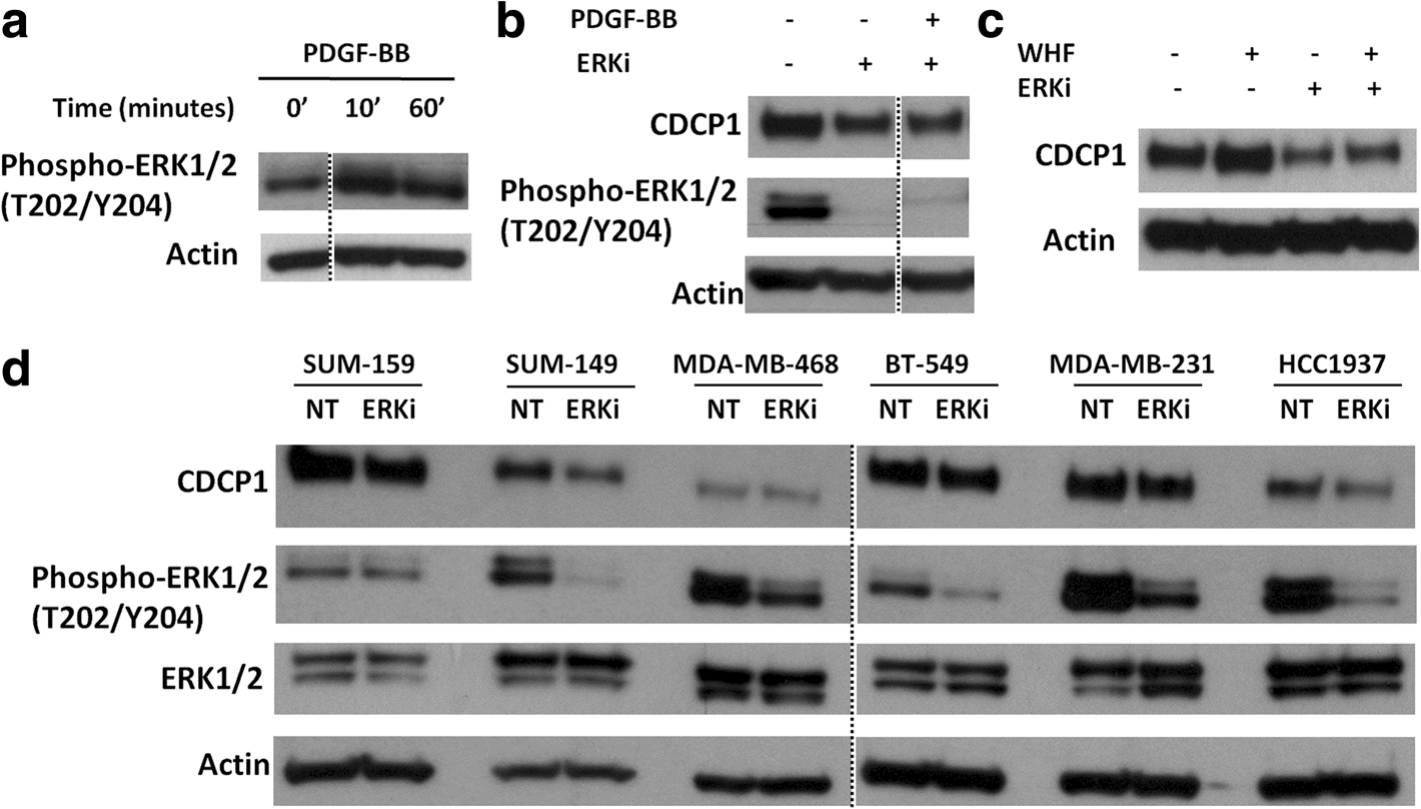
ERK1/2 activity regulates CDCP1 expression in TNBC cells. **a** WB analysis of phospho-ERK1/2 (T202/Y204) in MDA-MB-231 cells treated with or without 20 ng/ml PDGF-BB for 10 and 60 min. **b** WB analysis of CDCP1 and phosphoERK1/2 (T202/Y204) in MDA-MB-231 cells treated with or without the ERK1/2 inhibitor UO126 (2 μM) and stimulated with or without 20 ng/ml PDGF-BB for 24 h. **c** WB analysis of CDCP1 in MDA-MB 231 cells treated with or without UO126 (2 μM) and stimulated with or without 5% WHF in culture medium for 24 h. Dotted lines demarcate juxtaposed images originating from separate lines of the same western blot. **d** WB analysis of CDCP1, phosphoERK1/2 (T202/Y204), and ERK1/2 in SUM149, SUM159, MDA-MB468, BT-549, MDA-MB-231, and HCC1937 cells treated with or without UO126 (2 μM) under standard medium conditions for 24 h. Monoclonal anti-actin was used as the total protein loading control

To determine whether ERK activation is crucial in PDGF-BB-induced CDCP1 in our cell model, ERK status was examined on short-term stimulation, wherein downstream pathways of RTK are usually activated. MDA-MB-231 cells were starved and then treated with PDGF-BB for 10 and 60 min, confirming that PDGF-BB-induced PDGFRβ activation stimulated ERK1/2, starting from 10 min and persisting at 1 h (Fig. 3a).

To confirm that PDGF-BB stimulation induces CDCP1 expression through ERK1/2 activation, MDA-MB-231 cells were treated for 24 h with PDGF-BB with or without the ERK1/2 inhibitor UO126 (2 μM). ERK1/2 inactivation, on PDGF-BB treatment, mitigated the upregulation of CDCP1. CDCP1 levels fell in the presence of UO126 (Fig. 3b). Notably, UO126 diminished basal CDCP1 levels in unstimulated starved cells. CDCP1 upregulation upon the activation of the PDGFRβ/ERK axis was confirmed in two additionally triple negative breast cancer cell lines BT-549 and SUM149 (Additional file 2: Figure S2). These data indicate that PDGF-BB mediates the increase in CDCP1 through the ERK1/2 pathway.

Next, we starved MDA-MB-231 cells and treated them with or without a pool of 5 WHFs at a final concentration of 5% for 24 h, with or without UO126, to determine whether ERK1/2 activation is required for WHF-induced upmodulation of CDCP1 in TNBCs. By western blot, WHF increased CDCP1 levels only in the presence of functional ERK1/2 (Fig. 3c).

**Fig. 4:**
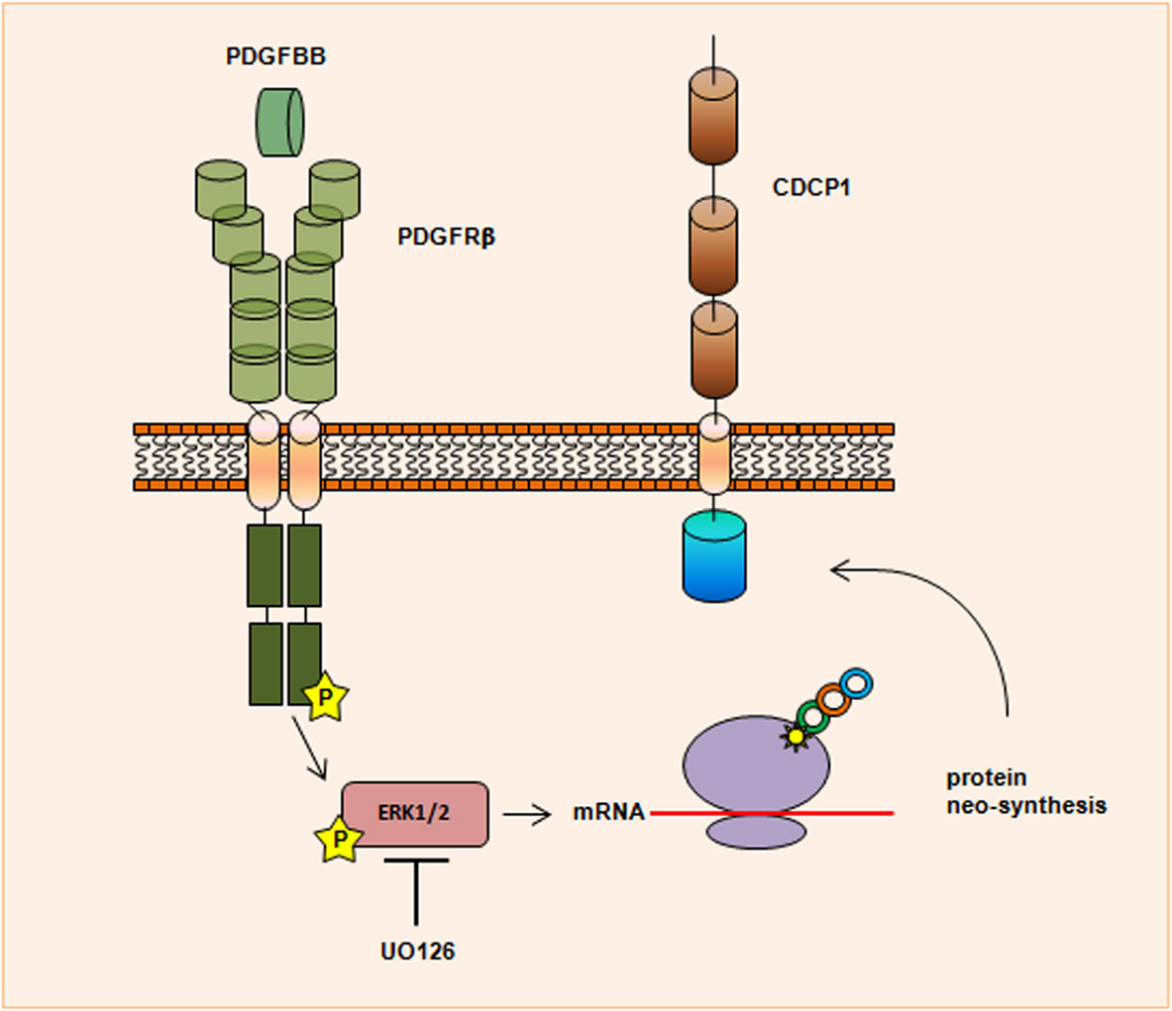
Schematic representation of CDCP1 upregulation induced by PDGF-BB/PDGFRβ pathway through ERK1/2 activation. PDGFRβ dimerizes and is activated upon binding of the PDGF-BB ligand, causing the activation of the kinase domain, visualized as tyrosine phosphorylation (P) of the receptor molecules. In conjunction with dimerization and kinase activation, the receptor molecules undergoes a conformational changes, which allow a basal kinase activity, leading to full enzymatic activity directed toward downstream mediators such as ERK1/2. ERK1/2 activity is necessary for CDCP1 protein neo-synthesis, as demonstrated by the reduction of CDCP1 protein levels in presence of UO126, an inhibitor of ERK1/2

To confirm that ERK1/2 is necessary for CDCP1 expression in TNBC cells, a panel of CDCP1-positive TNBC cell lines [3] was treated with UO126 under standard culture conditions for 24 h (Fig. 3d). CDCP1 levels decreased in 5 of the 6 cell lines on treatment with this ERK1/2 inhibitor; only in MDA-MB-468 cells CDCP1 expression was unaffected by ERK1/2 inactivation. These data confirm the implication of ERK1/2 in the regulation of CDCP1 expression in TNBCs. A schematic representation of CDCP1 upregulation upon PDGF-BB, PDGFRβ pathway activation, via ERK1/2 is shown in Fig. 4.

### CDCP1 and PDGFRβ expression associated in TNBC tissues

**Fig. 5:**
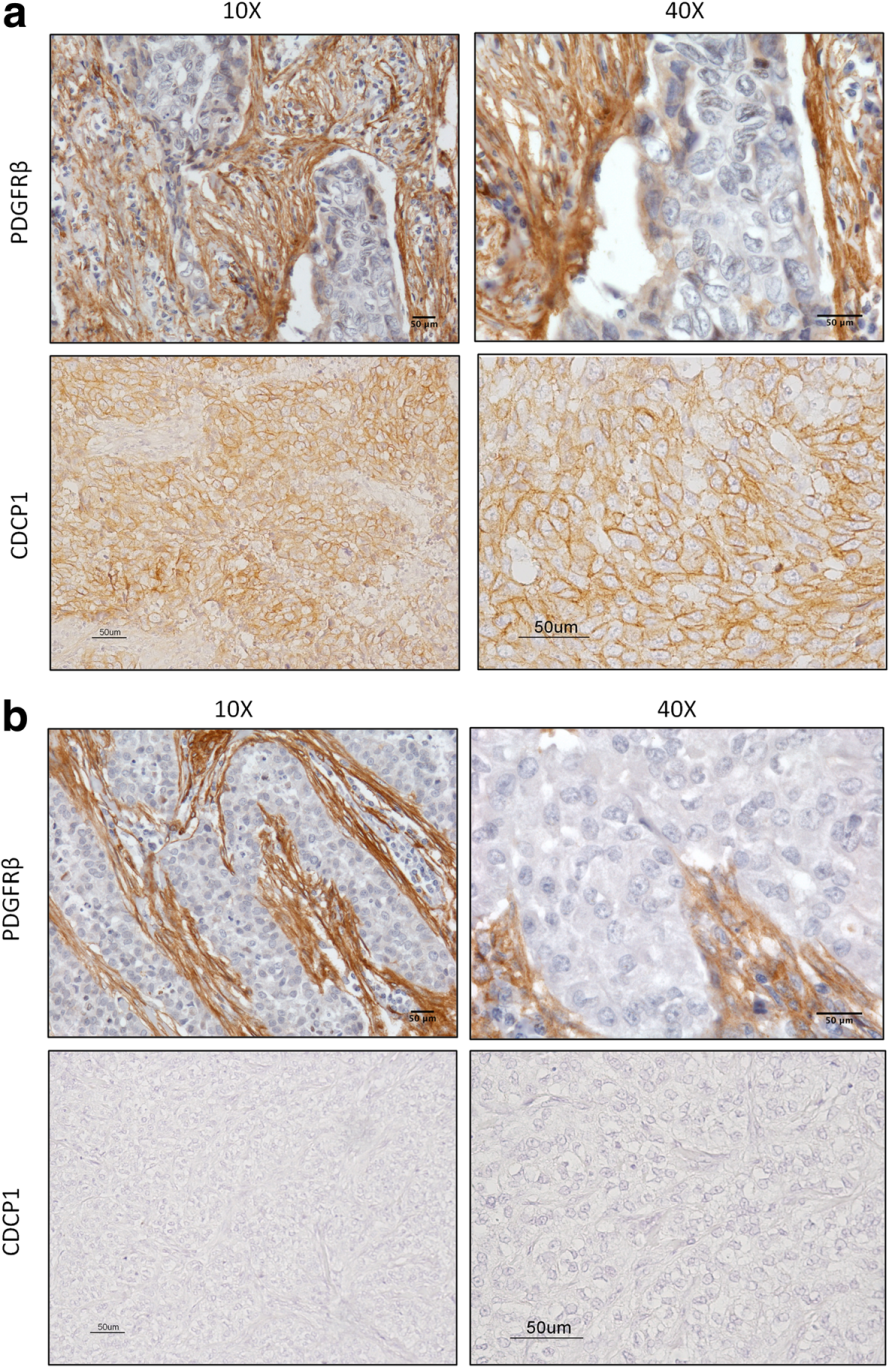
IHC staining of PDGFRβ and CDCP1 in TNBC. FFPE sections of TNBC specimens were analyzed by IHC for PDGFRβ and CDCP1. **a** Representative image of a PDGFRβ- and CDCP1-positive case, with plasma membrane staining, at 10X and 40X magnification; **b** Representative image of a PDGFRβ- and CDCP1-negative case, at 10X and 40X magnification

The association between PDGFRβ and CDCP1 expression was examined in 65 formalin-fixed, paraffin-embedded (FFPE) primary TNBC specimens by immunohistochemistry (IHC) (Table 1). Of these samples, 41.5% was node (N)-positive, and 60.0% of tumors were stage T1. As expected for TNBCs, the tumors were primarily grade III (84.6%) and highly necrotic (75.4%). Multifocality was observed in 19.9% of cases, and 35.4% of patients had ductal carcinoma in situ (DCIS). Tumors were categorized as CDCP1-positive or -negative per Turdo et al. [3] and were divided according to the presence or absence of PDGFRβ staining in association with tumor cells, as described in D’Ippolito et al. [25] (Fig. 5).

**Table 1:** Clinical characteristics of TNBC patients according to expression of PDGFRβ and CDCP1

	Overall cohort (*N* = 65^b^)	PDGFRβ pos (*N* = 29^b^)	PDGFRβ neg (*N* = 36^b^)	*P* value^c^	CDCP1 pos (*N* = 37^b^)	CDCP1 neg (*N* = 28^b^)	*P* value^c^
Age							
> = 50 years	42 (64.6%)	13 (44.8%)	29 (80.6%)	0,0040	22 (59.5%)	20 (71.4%)	0.4332
< 50 years	23 (35.4%)	16 (55.2%)	7 (19.4%)		15 (40.5%)	8 (28.6%)	
Grade							
I, II	9 (14.1%)	0 (0%)	9 (25.7%)	0,0029	5 (13.5%)	4 (14.8%)	1.0000
III	55 (85.9%)	29 (100.0%)	26 (74.3%)		32 (86.5%)	23 (85.2%)	
na	1		1			1	
Necrosis							
No	14 (22.2%)	6 (20.7%)	8 (23.5%)	1,0000	6 (16.7%)	8 (29.6%)	0.2395
Yes	49 (77.8%)	23 (79.3%)	26 (76.5%)		30 (83.3%)	19 (70.4%)	
na	2		2		1	1	
Multifocality							
No	52 (82.5%)	24 (82.8%)	28 (84.4%)	1,0000	28 (77.8%)	24 (88.9%)	0.3255
Yes	11 (17.5%)	5 (17.2%)	6 (17.6%)		8 (22.2%)	3 (11.1%)	
na	2		2		1	1	
N positivity							
No	38 (58.5%)	17 (58.6%)	21 (58.3%)	1,0000	19 (51.4%)	19 (67.9%)	0.2117
Yes	27 (41.5%)	12 (41.4%)	15 (41.7%)		18 (48.6%)	9 (32.1%)	
Size							
> 2 cm	39 (60.9%)	11 (39.3%)	15 (41.7%)	0,8035	15 (40.5%)	10 (37.0%)	0.8015
< = 2 cm	25 (39.1%)	18 (64.3%)	21 (58.3%)		22 (59.5%)	17 (63.0%)	
na	1	1				1	
DCIS^a^							
No	40 (63.5%)	20 (68.9%)	20 (58.9%)	0,4424	23 (63.9%)	17 (63.0%)	1.0000
Yes	23 (36.5%)	9 (31.0%)	14 (41.2%)		13 (36.1%)	10 (37.0%)	
na	2		2		1	1	

^a^DCIS, ductal carcinoma in situ

^b^Frequency percentages were calculated on available cases

^c^Fisher’s exact test

Because we have reported the function of PDGFRβ in mediating tumor vasculogenic properties in TNBCs [28], and we also reported that CDCP1 knocked-down impairs vasculogenic mimicry in vitro, TNBC tumors were then analyzed, based on the presence or absence of PDGFRβ staining in association with tumor cells in vascular-like structures or organized into tumor nests. Regarding tumor nests, 57% (37/65) of cases were CDCP1-positive, 45% (29/65) was PDGFRβ-positive, 32% (21/65) was positive for CDCP1 and PDGFRβ (double-positive), and 31% (20/65) was negative for CDCP1 and PDGFRβ (double-negative). CDCP1-positive cases did not differ significantly from their negative counterparts about clinical variables (Table 1), but PDGFRβ positivity was significantly associated with higher grade and younger age. All cases with grade I and II tumors did not express PDGFRβ, whereas nearly 50% of grade III tumors were PDGFRβ-positive (*p* = 0.0029; Fisher’s exact test); most PDGFRβ-positive cases were younger than PDGFRβ-negative cases (*p* = 0.0040; Fisher’s exact test).

About PDGFRβ expression in tumor and vascular lacunae according to CDCP1 levels, 72.4% (21/29) of PDGFRβ-positive cases in tumor nests were CDCP1-positive (Table 2; *p* = 0.0429; Fisher’s exact test). In our analysis of vascular lacunae, a trend of association was revealed between PDGFRβ in tumor cells in vascular-like structures and CDCP1 expression (Table 2; *p* = 0.0795; Fisher’s exact test).

**Table 2:** PDGFRβ expression in tumor and vascular lacunae according to expression of CDCP1 (IHC CDCP1)

	IHC CDCP1		*P* value^a^
	Pos	Neg	
PDGFRβ tumor nest			
Pos (*N* = 29)	21 (72.4%)^b^	8 (27.6%)	0.0429
Neg (*N* = 36)	16 (44.4%)	20 (55.5%)	
PDGFRβ vascular lacunae			
Pos (*N* = 27)	19 (70.4%)	8 (29.6%)	0.0795
Neg (*N* = 38)	18 (47.4%)	20 (52.6%)	

^a^Fisher’s exact test

**Fig. 6:**
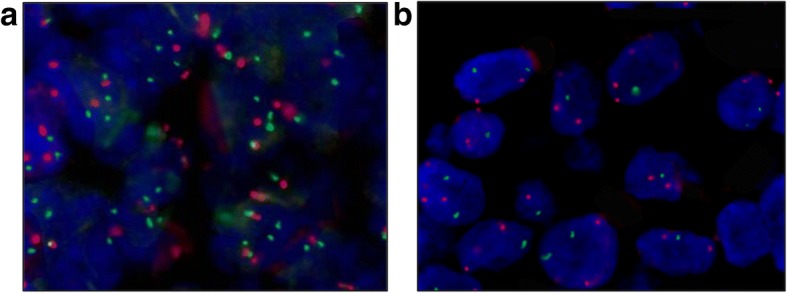
Genetic alterations of CDCP1 in TNBC. FFPE sections of TNBC specimens were analyzed by dual-color FISH using for CDCP1 genetic alteration CDCP1/CEP3 probes on a FFPE sections of TNBC specimens FISH. **a** Representative image of TNBC specimen positive for CDCP1 IHC staining, showing tumor cells with > 3 red signals for CEP3 and > 3 green signals for the CDCP1 locus (polysomy); **b** Representative image of TNBC specimen negative for CDCP1 IHC staining, showing tumor cells with > 3 signals for CEP3 and < 3 green signals for the CDCP1 locus (deletion)

Based on the relationship between CDCP1 expression and gains in *CDCP1*, we examined whether PDGFRβ was differentially expressed, depending on *CDCP1* status. *CDCP1* status was analyzed in 53 of 65 available TNBC specimens by fluorescence in situ hybridization (FISH). As a result, we identified 4 genetic categories: deleted (*n* = 2) and disomic (*n* = 40) cases were considered to be *CDCP1*-negative, and amplified (*n* = 2) and polysomic (*n* = 9) cases were *CDCP1-*positive (Fig. 6). Our FISH analysis did not reveal any imbalance in *CDCP1* gains in PDGFRβ-positive or -negative TNBC cases (Table 3). Among FISH-positive cases, 7 of 10 CDCP1-positive cases expressed PDGFRβ; similarly, among FISH-negative cases, 10 of 19 CDCP1-positive cases expressed PDGFRβ (*p* = 0.4495). These data demonstrate that CDCP1 and PDGFRβ expression is linked in TNBC specimens, independent of gains in *CDCP1*.

**Table 3:** PDGFRβ expression in tumor according with expression of CDCP1 protein (IHC CDCP1) and *CDCP1* genetic gain

		IHC CDCP1		
		Pos	Neg	*P* value^a^
*CDCP1* FISH neg	PDGFRβ tumor nest			
	Pos (*N* = 17)	10 (58.8%)	7 (41.2%)	0,2092
	Neg (*N* = 25)	9 (36.0%)	16 (64.0%)	
*CDCP1* FISH pos	PDGFRβ tumor nest			
	Pos (*N* = 7)	7 (100%)	0 (0%)	0.3636
	Neg (*N* = 4)	3 (75.0%)	1 (25.0%)	

^a^Fisher’s exact test

## Discussion

Our study described for the first time the role of PDGFRβ signalling in regulating CDCP1 expression in TNBCs. We have demonstrated that the induction of CDCP1 peaks on treatment with PDGF-BB, among various cytokines, chemokines, and growth factors, highlighting its significance in the regulation of this protein in the TNBC phenotype. Notably, the increase and maintenance of CDCP1 phosphorylation after treatment with PDGF-BB implicate PDGFRβ in the regulation of CDCP1 activity. Thus, we speculate that signals downstream of PDGFRβ are also crucial for the promotion of CDCP1-mediated prometastatic features through the activation of Src family kinases (SFKs). CDCP1 Tyr734 is the primary SFK-mediated phosphorylation site [13, 29], crucial for the recruitment of PKCδ and resulting in CDCP1-mediated invasiveness.

Growth factor receptors that are involved in tumor progression have been implicated in CDCP1 overexpression. The EGF-EGFR axis promotes CDCP1 expression in ovarian cancer models, and bone morphogenetic protein 4 induces CDCP1 in pancreatic cancer cells [5, 30]. Our analyses suggest that growth factors other than PDGF-BB mediate the upregulation of CDCP1. For example, HRG (likely via EGFR/HER3 heterodimerization) and FGF upmodulate CDCP1 on the cell membrane of TNBCs. The redundancy of signalling pathways that are downstream of these tyrosine kinase receptors suggests that the activation of several growth factor receptors converge at common mediators in CDCP1 synthesis, the most important of which are members of the RAS/RAF/MEK/ERK pathway, which have been implicated in CDCP1 mRNA and protein expression. Activation of Ras-ERK signalling alone induces CDCP1 expression in NCSLC, likely through the transcription factor AP-1 [21].

The RAS/RAF/ERK pathway is stimulated in TNBC [31], leading cells to acquire an aggressive phenotype—i.e., promoting invasiveness and migration [32]. Thus, ERK1/2 might also promote these features through the regulation of CDCP1 expression. Several stimuli, mediated by growth factors, converge to activate ERK1/2, paralleling the rise in CDCP1 on the treatment of MDA-MB-231 cells with the various growth factors that we tested. As observed in a panel of TNBC models, treatment with an ERK1/2 inhibitor under standard culture conditions downregulated CDCP1, demonstrating that this pathway is crucial for CDCP1 expression in the TNBC phenotype. The MDA-MB-468 cell line was the only model that showed no variation in the CDCP1 expression on ERK1/2 inhibition; similarly, it was the TNBC line that upregulated CDCP1 RNA and protein the least on treatment with WHD [3]. These data suggest the existence of another regulatory mechanism in addition to RTK/ERK1/2 activation. That ERK is less active when the PDGFRβ expression is abrogated in TNBC confirms the link between these two tyrosine kinases. CDCP1 expression regulation, upon PDGFRβ/ERK1/2 pathway activation, was investigated also in non-breast cancer cells. CDCP1 expression did not increase upon PDGF-BB treatment in the human large cell lung cancer cells NCI-H460 (ATCC® HTB-177™), whereas it was slightly up-regulated in the human esophageal adenocarcinoma cells OE19 (Sigma-Aldrich). Interestingly, in both cell lines, the presence of ERK1/2 inhibitor strongly reduces CDCP1 protein level (unpublished data). This data suggests that ERK1/2 could be a crucial hub for the regulation of CDCP1 expression, not only in breast cancer cells. As a support, it has been shown that CDCP1 expression is regulated through ERK1/2 recruitment in ovarian cancer cells stimulated with EGF [29]. On the contrary, the sensitivity to PDGFRβ activation seems to be dependant by the cells.

The PDGFRβ axis is involved in breast cancer because tumor tissue and the surrounding stroma express PDGFRβ [33, 34]. Stromal PDGFRβ expression is associated with a poor prognosis [35, 36]. Also, breast cancer cells and fibroblasts secreted PDGF-like factors that sustain the PDGFR pathway in tumor cells [37, 38]. Considering the low expression of PDGFRα in MDA-MB-231 cells [39], we cannot exclude that other isoforms of PDGF receptors regulate CDCP1 expression. Notably, by immunohistochemistry, PDGFRβ and CDCP1 expression correlated significantly in a cohort of 65 TNBC specimens, confirming that the PDGF-BB/PDGFRβ axis governs CDCP1 expression in human tumors.

However, supporting that several growth factor receptors can regulate the expression of CDCP1, not all CDCP1-positive specimens expressed PDGFRβ in the tumor cells. We have reported that gains in *CDCP1* are significantly associated with CDCP1 expression but that nearly half of CDCP1-positive cases do not show such gains. In the current study, in the absence of a gain in *CDCP1*, CDCP1 was expressed at the same frequency in PDGFRβ-positive and -negative TNBC specimens, indicating that PDGFRβ supports CDCP1 expression independently of a gain in *CDCP1*. Accordingly, *CDCP1* polysomy was observed in MDA-MB-231 cells (data not shown), in which PDGFRβ stimulation further increased basal CDCP1 levels.

Further, our group has demonstrated that the ability of TNBCs to form vascular-like channels [28] is associated with increased tumor aggressiveness and that this phenotype is related strictly to the expression of PDGFRβ [25]. Considering that CDCP1 also contributes to vasculogenic mimicry [3], we hypothesize that PDGFRβ mediated this peculiar TNBC phenotype by regulating the expression of CDCP1. Accordingly, the association between PDGFRβ and CDCP1 was nearly significant—almost 70% of vascular lacunae that expressed PDGFRβ were positive for CDCP1.

In our previous paper on CDCP1 role in TNBC [3], we showed that knock-down of CDCP1 expression in TNBC cell lines did not affect their in vitro growth capability in 2D cultures and, accordingly, no association was found between CDCP1 expression and proliferation rates in TNBC specimens, evaluated by Ki-67 marker. Regarding the role of PDGFRβ, it is crucial for the vasculogenic properties of tumor cells, and therefore, its role in tumorigenesis mainly accounts for the activation of migration/invasion/angiogenesis pathways in cancer cells. Consistently, in a previous paper [27], we reported that inhibition of PDGFRβ pathways only slightly influences proliferation of TNBC, and that its role in TNBC aggressiveness mainly depends on the capacity to induce vasculogenic mimicry.

In conclusion, we have identified PDGF-BB/PDGFRβ as a new pathway that is involved in the regulation of CDCP1 expression in TNCBs through ERK1/2 activation. Our results provide the basis for the potential use of PDGFRβ and ERK1/2 inhibitors in targeting the high aggressiveness of TNBCs.

## Conclusions

We have identified PDGF-BB/PDGFRβ–mediated pathway as a novel player in the regulation of CDCP1 in TNCBs through ERK1/2 activation. Our results provide the basis for the potential use of PDGFRβ and ERK1/2 inhibitors in targeting the aggressive features of CDCP1-positive TNBCs.

## Additional files

Additional file 1: **Figure S1.** Gating strategy for CDCP1 flow cytometric analysis. Flow cytometric analysis of MDA-MB-231 cells starved in serum-free medium for 24 h and then treated for 48 h with FGF 50 ng/mL. (PDF 470 kb)

Additional file 2: **Figure S2.** PDGFR-BB stimulation upregulates CDCP1 in TNBC cells. Western blot analysis of CDCP1 and Vinculin expression in SUM-149 and BT549 cells upon PDGF-BB and ERKi treatment. (PDF 249 kb)

## Abbreviations

CDCP1: CUB domain-containing protein-1
ERK1/2: Extracellular signal-regulated kinases 1–2
FISH: Fluorescent in situ hybridization
IHC: Immunohistochemistry
PDGF-BB: Platelet-derived growth factor-BB
PDGFRβ: Platelet-derived growth factor receptors beta
TNBC: Triple-negative breast cancer
WHFs: Wound Healing Fluids
