# Supplementary material for: The PDGFRβ/ERK1/2 pathway regulates CDCP1 expression in triple-negative breast cancer
Source: BMC Cancer. 2018 May 23;18:586. doi: 10.1186/s12885-018-4500-9 (PMC5967041; doi:10.1186/s12885-018-4500-9)
Supplement: Supplementary file 1 — Figure S1. Gating strategy for CDCP1 flow cytometric analysis. Flow cytometric analysis of MDA-MB-231 cells starved in serum-free medium for 24 h and then treated for 48 h with FGF 50 ng/mL. (PDF 470 kb) [file 12885_2018_4500_MOESM1_ESM.pdf]

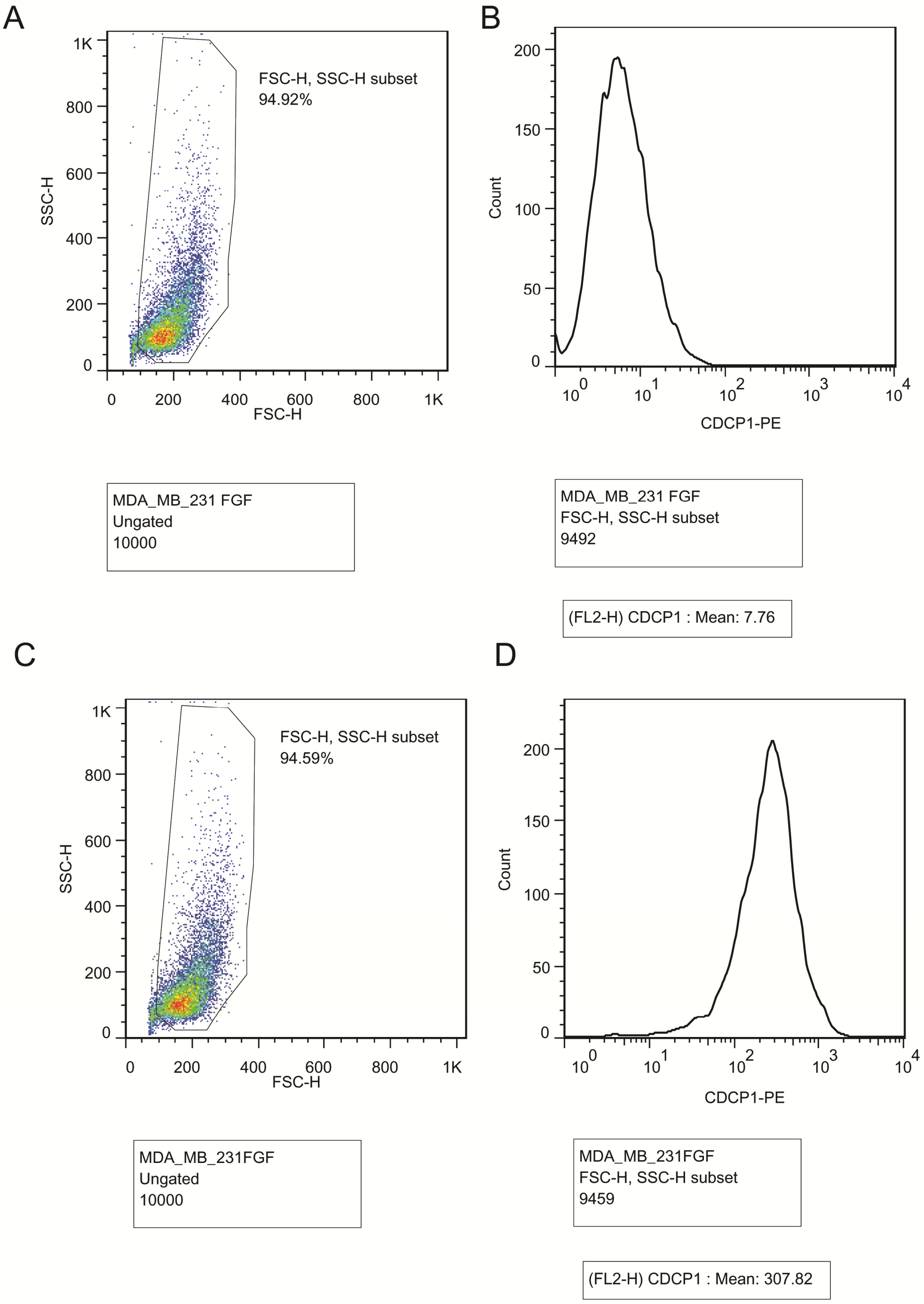

Figure S1. Gating strategy for CDCP1 flow cytometric analysis. Rapresentative images of FGF stimulation of MDA-MB-231 cells. MDA-MB-231 cells were starved in serum-free medium for 24 h and then treated for 48 h with FGF 50 ng/mL (PeproTech). Cells were harvested with Versene (Lonza) and wash with PBS 1X. CDCP1 protein was detected by FACScan analysis by staining cells with PE anti-human CD318 (CDCP1) Antibody (BioLegends). Cells not stained with antibody were used as controls. 10000 events was acquired. The gates were set based on light scatter properties after debris and doublet exclusion. Samples were analyzed using a FACSCalibur flow cytometer (BD Biosciences) and FlowJo software (TreeStar). Dot plot (A) and histogram plot (B) of MDA-MB-231 cells not stained, used as control; Dot plot (C) and histogram plot (D) of MDA-MB-231 cells stained with anti-CDCP1.
