# Supplementary material for: The PDGFRβ/ERK1/2 pathway regulates CDCP1 expression in triple-negative breast cancer
Source: BMC Cancer. 2018 May 23;18:586. doi: 10.1186/s12885-018-4500-9 (PMC5967041; doi:10.1186/s12885-018-4500-9)
Supplement: Supplementary file 2 — Figure S2. PDGFR-BB stimulation upregulates CDCP1 in TNBC cells. Western blot analysis of CDCP1 and Vinculin expression in SUM-149 and BT549 cells upon PDGF-BB and ERKi treatment. (PDF 249 kb) [file 12885_2018_4500_MOESM2_ESM.pdf]

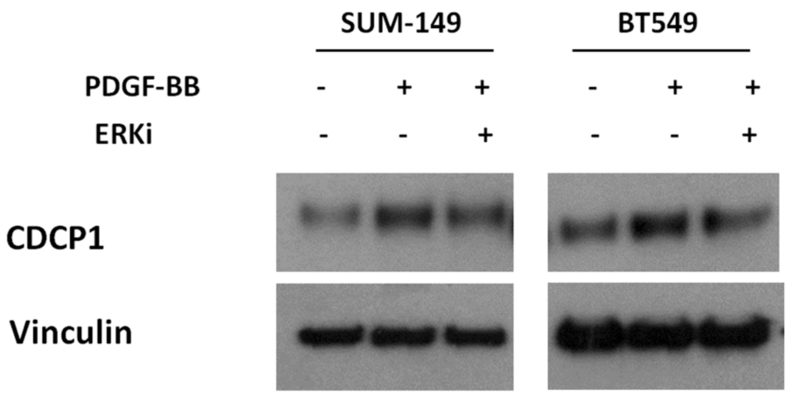

Supplementary Fig. 2. PDGFR-BB stimulation upregulates CDCP1 in TNBC cells. WB analysis of CDCP1 in SUM-149 and BT549 cells treated with or without the ERK1/2 inhibitor UO126 (2 µM) and stimulated with or without PDGF-BB 20 ng/ml for 48 h. Monoclonal anti-vinculin antibody was used as the total protein loading control.
